# Socioeconomic Inequalities in the Mortality of Hodgkin and Non-Hodgkin Lymphoma: A Two-Decade Trend Analysis

**DOI:** 10.3390/curroncol33080470

**Published:** 2026-08-07

**Authors:** Kelly Zhang, Ali Kiadaliri, Mohammad Hajizadeh

**Affiliations:** 1 Faculty of Medicine, Dalhousie University, Halifax, NS B3H 4R2, Canada; 2 Department of Clinical Sciences–Lund, Orthopaedics, Lund University, SE-221 85 Lund, Sweden; 3 Division of Insurance Medicine, Department of Clinical Neuroscience, Karolinska Institute, SE-171 77 Stockholm, Sweden; 4 School of Health Administration, Faculty of Health, Dalhousie University, Halifax, NS B3H 4R2, Canada

**Keywords:** Hodgkin and non-Hodgkin lymphoma, income, education, inequalities, Canada

## Abstract

Lymphomas are broadly categorized into Hodgkin lymphoma (HL) and non-Hodgkin lymphoma (NHL). Despite treatment advances, they remain a major cause of cancer-related morbidity and mortality in Canada. This study examined temporal trends in socioeconomic inequalities in lymphoma mortality in Canada from 2000 to 2019. Using a unique census division level dataset (n = 280) constructed by pooling information from the Canadian Vital Statistics Death Database, the Canadian Census of Population and the National Household Survey, we measured mortality in HL and NHL in Canada. The age-standardized Concentration index (C) was used to quantify income and education inequalities in lymphoma. Time trend analyses were conducted to examine the changes in the observed socioeconomic inequalities. Crude HL mortality declined significantly over the study period. Crude NHL mortality remained largely unchanged in Canada overall, although modest declines were observed in the Prairies. The age-standardized C indicated persistent income and education inequalities for both lymphoma types. For NHL, mortality became increasingly concentrated among lower-income and lower-education populations over time. The persistent and widening socioeconomic inequalities observed in HL and NHL mortality, respectively, in Canada underscore the need for targeted public health interventions and more equitable distribution of resources to address systematic and avoidable differences in cancer outcomes associated with socioeconomic disadvantage.

## Simple Summary

Despite significant medical advancements, lymphomas remain a leading cause of cancer-related illness and death in Canada. While mortality from Hodgkin lymphoma has generally decreased, deaths from non-Hodgkin lymphoma did not decline in Canada. Importantly, this study shows that these deaths are not distributed equally; individuals with lower incomes and less education face a higher risk of dying from these cancers. These disparities have increased over time, with the burden of non-Hodgkin lymphoma increasingly concentrated among the most socioeconomically disadvantaged populations. These findings suggest that medical breakthroughs alone are not sufficient to ensure survival for everyone. To improve outcomes, future health policies should focus on reducing these systemic barriers by ensuring that life-saving cancer treatments and early detection programs are equally accessible to all Canadians, regardless of socioeconomic status.

## 1. Introduction

Lymphomas are a heterogeneous group of hematologic malignancies that originate in the lymphatic system and are broadly classified into Hodgkin lymphoma (HL) and non-Hodgkin lymphoma (NHL). In Canada, lymphoma is a leading cause of cancer-related morbidity and mortality, affecting patients across all age groups and placing considerable demands on healthcare resources. In 2026, an estimated 12,100 Canadians were diagnosed with NHL, and approximately 3200 Canadians died from it [1]. HL, on the other hand, was diagnosed in 1250 Canadians, with about 120 deaths [2].

NHL is a broad umbrella term encompassing various types of lymphoid malignancies. Common subtypes include follicular lymphoma, a generally indolent lymphoma characterized by a prolonged disease course, and diffuse large B-cell lymphoma (DLBCL), a more aggressive subtype requiring prompt treatment. It can be further classified into B-cell, T-cell, and natural killer (NK) cell malignancies based on the type of lymphocyte affected [3,4]. By contrast, HL is distinctively characterized by the presentation of Reed-Sternberg cells, with higher cure rates due to the assistance of chemotherapy and radiotherapy.

The epidemiology of HL and NHL differs substantially. HL demonstrates a bimodal age distribution, with incidence peaks occurring in young adulthood and older adults, whereas NHL occurs predominately among older adults [5]. Males generally experience higher incidence rates than females for both HL and NHL, although patterns vary across specific subtypes [6]. Because HL is more common among younger individuals and is associated with relatively higher long-term survival, mortality rates are comparatively low [7,8]. NHL accounts for the majority of lymphoma-related deaths due to its greater incidence and poor prognosis associated with more aggressive subtypes [9].

The development of lymphoma is influenced by a combination of genetic, immunologic, environmental, and lifestyle factors. Many of these risk factors are non-modifiable. Individuals with a familial predisposition, immune system impairment (such as autoimmune disorders and immunosuppression in cancer treatments), or viral exposures, particularly Epstein–Barr virus (EBV) infection, are at greater risk of developing both HL and NHL. Other infectious agents, including human immunodeficiency virus (HIV) and human T-cell lymphotropic virus type 1 (HTLV-1), have also been associated with lymphoma development. Environmental exposures to pesticides, organic solvents, and ionizing radiation have been implicated as possible contributors. Some studies further suggest a modest association between lifestyle factors such as smoking, obesity, and diet and the incidence of lymphoma, although these relationships are less consistent across populations [10].

Socioeconomic status (SES) has been recognized as an important determinant of cancer outcomes, including mortality [11,12,13,14,15,16]. Socioeconomic inequalities in cancer mortality may arise through differences in environmental exposures, health behaviors, access to healthcare services, early diagnosis, treatment utilization, and survival outcomes [17,18,19]. In lymphoma, previous studies investigating socioeconomic differences in outcomes have reported mixed findings. Some studies suggest that individuals with higher SES experience better lymphoma survival outcomes, potentially due to improved access to healthcare and earlier diagnosis [20,21]. In contrast, other studies have found limited or no association between SES and survival among patients with certain lymphoma subtypes, such as diffuse large B-cell lymphoma [22,23]. While these studies provide important insights into socioeconomic differences in lymphoma outcomes, considerably less is known about socioeconomic inequalities in lymphoma mortality at the population level, particularly in Canada. Examining mortality provides an important population health perspective because mortality reflects both disease occurrence and survival outcomes and may therefore capture broader socioeconomic inequalities in lymphoma burden.

Existing studies from several countries suggest that socioeconomic disadvantage may be associated with higher lymphoma mortality rates, although findings have been inconsistent and may vary according to lymphoma subtype, sex, age, healthcare system, and socioeconomic indicator used [6,21,24,25,26,27]. To our knowledge, no pan-Canadian study has comprehensively examined socioeconomic inequalities in HL and NHL mortality over time. Therefore, this study investigates the relationship between SES and lymphoma mortality in Canada between 2000 and 2019, a period corresponding to the widespread adoption of rituximab in the treatment of B-cell lymphomas, thereby providing a more clinically comparable timeframe for examining mortality inequalities. The analysis excluded the territories (Yukon, Northwest Territories, and Nunavut), which together account for 0.03% of the total population [28]. A better understanding of this association may help inform public policies and healthcare strategies aimed at reducing inequalities in lymphoma outcomes across SES groups.

## 2. Methods

### 2.1. Data and Variables

This study is based on dataset compiled at the level of Census Divisions (CD, n = 280) in Canada, using data from the Canadian Vital Statistics Death Database (CVSD), the 2011 National Household Survey (NHS), and multiple cycles of the Canadian Census of Population (CCP, 2001, 2006, and 2016). A CD is a geographic area defined by Statistics Canada for regional planning and the delivery of administrative services, representing a region between the provincial/territorial and municipal levels [29]. The CVSD records the number of deaths that occur in Canada each year as reported by the provincial and territorial Vital Statistics Registries to Statistics Canada, which also provides demographic information and cause of death [30]. The CVSD was used to identify individuals whose death was caused by lymphoma. Using the International Classification of Diseases (ICD) versions 10, HL was identified using ICD-10 code C81. NHL was identified using ICD-10 codes C82–C85, and C96.3 [31]. Data on sex and age distribution at the CD level were attained from the CCP and NHS. The CCP collects information on Canadian population, including Canadian citizens, landed immigrants, and non-permanent residents and their families living in Canada, and collects detailed socioeconomic and sociodemographic information [32]. Conducted every five years, the CCP data for the two years before and after each Census cycle were linked with HL and NHL mortality data. The CCP includes both a mandatory “short-form Census,” completed by all households, and historically a mandatory “long-form Census,” completed by approximately 20–25% of households and containing nearly 60 questions. In 2011, the mandatory long-form Census was replaced by the voluntary NHS, which included questions nearly identical to those in the previous long-form Census. To maintain representativeness, approximately 33% of Canadian households were selected to participate in the NHS. Because the short-form Census does not include SES information, data from the 2011 NHS were used to obtain SES measures for the 2009–2013 period. The NHS provides important demographic, and socioeconomic that supports government planning and resource allocation in Canada [33].

First, the number of deaths attributed to HL and NHL between 2000 and 2019 was obtained from the CVSD for each CD. The CCP and NHS data for the corresponding periods and CDs were used to obtain SES and demographic information, including the average age of females and the female population size, for each CD. We measured and used three SES measures at the CD: mean household income, median household income, and educational attainment (measured as the proportion of the population with a bachelor’s degree or higher). To measure mean and median household income, we adjusted household income for household size using the Organization for Economic Co-operation and Development (OECD) equivalence scale, calculated by dividing household income by the square root of household size [34]. The demographic and socioeconomic information obtained for each CD was linked to the corresponding mortality data to measure socioeconomic inequalities in lymphoma cancer mortality over time.

### 2.2. Statistical Analysis

#### 2.2.1. Measuring Socioeconomic Inequalities

We used the Concentration index (C) to quantify income- and education-related inequalities in HL and NHL mortality in Canada. The C is derived from the concentration curve (CC), which plots the cumulative proportion of the population ranked by SES (income or education in this study) on the horizontal axis against the cumulative proportion of lymphoma mortality on the vertical axis. If the CC coincides with the 45-degree line of equality (the line of perfect equality), this indicates no socioeconomic inequality in mortality. A CC lying below the line of equality indicates that lymphoma mortality is disproportionately concentrated among lower-SES population groups, whereas a CC lying above the line indicates concentration among higher-SES groups. The C is defined as twice the area between the concentration curve and the line of perfect equality.

The C can be calculated using the “convenient regression” formula [35]:

(1) $$2\sigma_{R}^{2}\left(\frac{{Ly}_{i}}{M}\right)=\alpha +\beta R_{i}+\varepsilon_{i},$$

where $\sigma_{R}^{2}$ denotes the variance of the fractional rank of SES; *i* indexes the CD; ${Ly}_{i}\;$ represents HL or NHL mortality in CD *i*; M is the mean HL (or NHL) mortality rate across all CDs; α is the intercept; β is the slope coefficient (the ordinary least squares [OLS] estimate of *β* and its standard error are equivalent to the value and standard error of the crude C); $R_{i}$ is the weighted fractional rank of CD *i*—weighted by each CD’s share of the total Canadian population—in the SES distribution, calculated as i/n, where i = 1 and i = n correspond to the lowest- and highest-SES CDs, respectively; and $\varepsilon_{i},$ is the error term [36].

Because cancer mortality varies substantially by age, we applied indirect age standardization to the convenient regression formulation to ensure that the concentration index reflects the socioeconomic distribution of HL (or NHL) mortality independent of differences in age composition across CDs, as follows:

(2) $$2\sigma_{R}^{2}\left(\frac{{Ly}_{i}}{M}\right)=\alpha +{\gamma R}_{i}+{Age}_{i}+\varepsilon_{i},$$

where γ indicates the slope (equivalent to the age-standardized C); and ${Age}_{i}$ represents the average age of the ith CD. The standard error derived from γ in Equation (2) corresponds to the standard error of the age-standardized C [35]. For each year, three age-standardized Cs were estimated, each corresponding to a different SES measure: mean equivalized household income, median equivalized household income, and educational attainment. While for NHL, analyses stratified by sex were conducted, we were not able to do sex-stratified analyses for HL due to the small HL-related deaths.

#### 2.2.2. Trend Analyses

Linear trend analyses were conducted to assess temporal trends in lymphoma mortality (HL or NHL) and socioeconomic inequalities in lymphoma mortality. Specifically, regression models were estimated with either crude lymphoma mortality rates or age-standardized C as the dependent variables, depending on whether the focus was on mortality trends or socioeconomic inequalities, respectively, and calendar year as the independent variable. A statistically significant positive (negative) trend coefficient for crude mortality rates indicates increasing (decreasing) mortality over time. A positive (negative) trend coefficient for the age-standardized C indicates a growing concentration of lymphoma mortality among higher-SES (lower-SES) groups, as defined by income or educational attainment. Statistical significance was assessed at *p* < 0.05. All analyses were conducted using Stata version 18.0 (College Station, TX, USA).

## 3. Results

### 3.1. Trends in the Mortality of HL and NHL

Table 1 and Table 2 present crude HL and NHL mortality rates per 100,000 population for both sexes by Canadian region from 2000 to 2019, respectively. Mortality data for both HL and NHL were grouped by geographic region: British Columbia; the Prairies (Alberta, Saskatchewan, and Manitoba); Ontario; and Eastern Canada (Quebec, Nova Scotia, New Brunswick, Newfoundland and Labrador, and Prince Edward Island).

As reported in Table 1, there was a statistically significant decline in crude HL mortality across Canada, as indicated by the negative trend coefficient (−0.005). Similar declines were observed in the Prairies and Ontario. British Columbia and Eastern Canada were the regions that did not exhibit a statistically significant decline in crude HL mortality.

Table 2 presents crude NHL mortality rates per 100,000 population by Canadian region from 2000 to 2019, shown for both sexes combined as well as separately for males and females. Nationally, NHL mortality remained relatively stable over the study period. However, the Prairies were the only region to experience an overall decline in NHL mortality among both sexes combined. Among females, NHL mortality declined over time in Canada overall, with statistically significant reductions observed in the Prairies and Ontario. In contrast, male NHL mortality remained stable nationally. Eastern Canada was the only region to experience a statistically significant increase in male NHL mortality, while the Prairies showed a significant decline.

### 3.2. Socioeconomic Inequalities in the Mortality of HL and NHL

**Figure 1:**
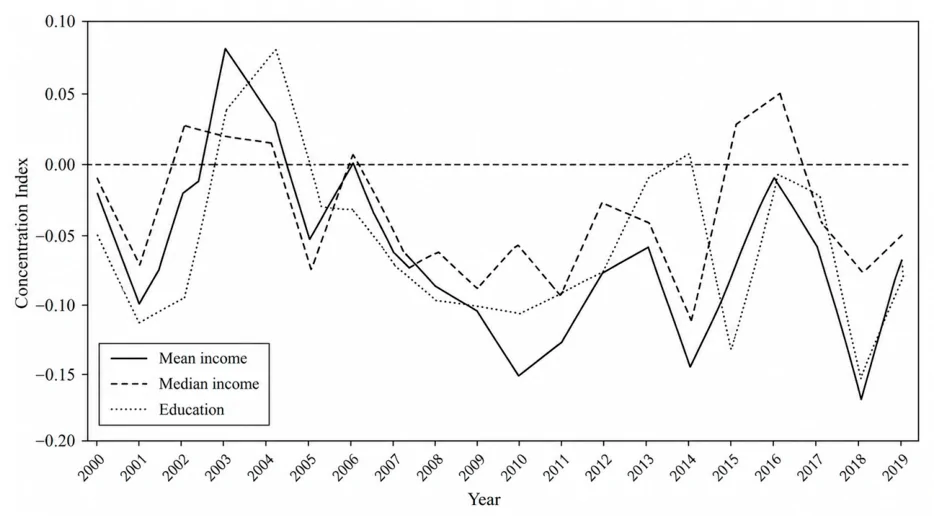
Income and education inequalities in Hodgkin lymphoma mortality for both sexes in Canada: 2000–2019.

Table 3 reports socioeconomic inequalities in HL mortality using age-standardized C based on the three SES measures, with the corresponding trends illustrated in Figure 1. The age-standardized C was consistently negative in most years, indicating that HL mortality was disproportionately concentrated among CDs with lower SES. These negative values reached statistical significance in several years, including 2001, 2008, 2010, 2011, 2014, 2015, and 2018. Trend analyses did not indicate any significant changes in income- or education-related inequalities in HL mortality over the study period.

**Figure 2:**
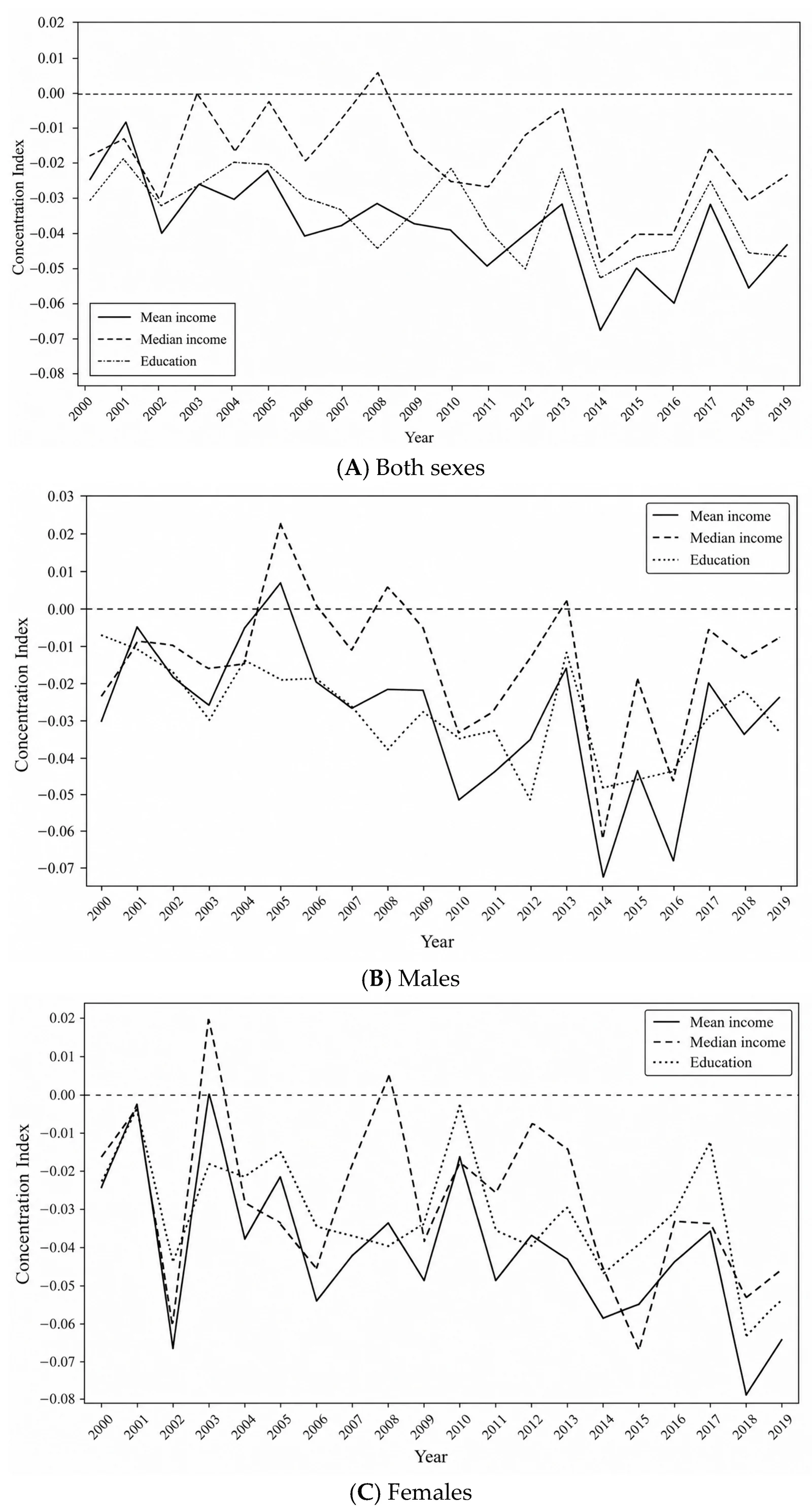
Income and education inequalities in non-Hodgkin lymphoma mortality for both sexes, males, and females in Canada: 2000–2019.

Table 4 reports age-standardized C for NHL mortality in Canada from 2000 to 2019 based on mean and median equivalized household income, disaggregated by sex. Figure 2 illustrate the corresponding values of the C. The consistently negative values of the C indicate that NHL mortality over this period was disproportionately concentrated among CDs with lower incomes for both males and females. These negative values reached statistical significance in more recent years, particularly when SES was measured using mean equivalized household income. Overall, females exhibited stronger and more frequent evidence of income-related inequality in NHL mortality than males, as reflected by C values with greater negative magnitude compared with their male counterparts. Trend analyses indicated a significant increase in the concentration of NHL mortality among lower SES groups over the study period, particularly when mean equivalized household income was used as the SES measure.

Table 5 reports age-standardized C for NHL mortality in Canada from 2000 to 2019 based on education level, disaggregated by sex. Figure 2 illustrates the corresponding values of the C. The negative values of the C suggest that NHL mortality in Canada was consistently higher among CDs with less education, particularly among females. Statistically significant education-related inequalities were observed in approximately one-third of the study years among males and females. Trend analyses indicated that education-related inequality in NHL mortality increased over the study period.

## 4. Discussion

In this study, we assessed long-term trends in socioeconomic inequalities, focusing on income- and education-related inequalities in HL and NHL mortality in Canada between 2000 and 2019. We first discuss patterns in crude HL and NHL mortality over time, as crude mortality rates are less readily available than age-standardized rates and reflect the actual burden of mortality in the population, making them particularly useful for healthcare planning and resource allocation. We then interpret age-standardized income and education inequalities in lymphoma mortality.

### 4.1. Trends in HL and NHL Mortality

This study found a statistically significant decline in crude HL mortality rates across Canada from 2000 to 2019. This decline was particularly evident in the Prairies and Ontario, whereas other regions did not exhibit a statistically significant change throughout the study period. Because these findings are based on crude mortality rates, they should be interpreted with caution, as temporal trends may also reflect changes in the population age structure over time. Nevertheless, the observed reduction in crude HL mortality in Canada is consistent with improvements in HL management and may partly reflect advances in treatment, including the introduction of novel therapies such as brentuximab vedotin, initially used for relapsed or refractory disease and later in consolidation settings. These advances have reduced treatment-related toxicity, improved salvage outcomes, and lowered relapse risk following transplantation [37]. In addition, the adoption of positron emission tomography–computed tomography (PET-CT) for staging and PET-adapted treatment strategies has enabled more tailored therapy intensity, contributing to improved cure rates while minimizing long-term toxicity [38].

Unlike HL mortality, crude NHL mortality exhibited a different pattern over time. Between 2000 and 2019, national crude NHL mortality did not change in Canada, although the Prairies showed a decline in crude NHL mortality. Sex-stratified analyses further highlighted these patterns: among males, a statistically significant increase was observed only in Eastern Canada, while the Prairies showed a significant decline; among females, crude NHL mortality declined significantly at the national level and in both the Prairies and Ontario, with no statistically significant changes in Eastern Canada.

These divergent trends may be partly attributable to the biological and epidemiological heterogeneity of NHL. NHL comprises multiple subtypes with distinct incidence trends, age distributions, and treatment responses. However, because these findings are based on crude mortality rates, the observed temporal trends may reflect changes in both age-specific mortality and the underlying demographic structure of the population. In particular, Canada’s ageing population may have influenced the observed NHL mortality trends, given the substantially higher incidence and mortality of NHL among older adults. In Canada, the population aged 85 years and older has doubled since 2001, with Quebec having the highest proportion of individuals in this age group, followed by New Brunswick, Saskatchewan, British Columbia, Ontario, Nova Scotia, Prince Edward Island, Manitoba, Newfoundland and Labrador, and Alberta [39]. This demographic pattern may have contributed to the observed regional variation in crude NHL mortality, including the decline observed in the Prairies, where provinces such as Manitoba and Alberta have among the lowest proportions of individuals aged 85 years and older [39]. The more pronounced and widespread increases in crude NHL mortality among males, particularly in Eastern Canada, alongside a contrasting decline among females in multiple regions, may also reflect differences in subtype distribution or greater occupational exposure to carcinogens such as pesticides, benzene, and trichloroethylene, which are more prevalent in male-dominated occupations and have been linked to more aggressive NHL subtypes [40].

### 4.2. Trends in Socioeconomic Inequalities in HL and NHL Mortality

The association between SES and lymphoma outcomes is well documented globally. Individuals from lower SES backgrounds are more likely to delay seeking primary care, which can postpone access to diagnostic procedures such as imaging and biopsy and ultimately lead to poorer prognoses [41,42,43]. In addition, the high cost of cancer treatment can impose substantial indirect financial strain. Lost wages, childcare responsibilities, and transportation costs can disproportionately affect these patients.

In our study, using age-standardized C, HL mortality was consistently concentrated in lower-income groups, with no evidence that income-related inequalities changed over time. These patterns are notable given HL’s overall curability and imply that social determinants, rather than biology alone, are driving persistent mortality gaps. Several hypotheses may explain the observed inequalities. Likely mechanisms include differential access to timely diagnosis, variation in comorbidity burden and frailty that complicate HL treatment, and barriers to completing curative-intent therapy (e.g., transportation, paid sick leave, caregiver availability). With respect to education, lower health literacy and delays in primary-to-specialist referral may also contribute. Importantly, median and mean income measures may capture different aspects of material resources; the consistently negative C values strengthen the inference that material disadvantage concentrates HL mortality.

Similar to HL mortality, NHL mortality remained disproportionately concentrated among lower-income and less-educated populations in Canada, with stronger and more consistent evidence observed among females. Several mechanisms may link lower SES to higher NHL mortality. Lower educational attainment may reduce health literacy and hinder navigation of complex cancer care pathways, while also correlating with occupational exposures and comorbidities that worsen prognosis. Income disadvantage may further constrain access to specialty care, limit the ability to travel for multi-day infusions, and reduce access to supportive care resources.

Unlike HL, we observed increasing income and education inequalities in NHL over the study period. The widening of these inequalities suggests that improvements in NHL outcomes may not have been experienced equally across socioeconomic groups. Although Canada has a universal healthcare system and therapeutic advances, including rituximab, have substantially improved NHL survival, universal healthcare does not necessarily eliminate socioeconomic inequalities in cancer outcomes. Socioeconomically disadvantaged individuals may continue to face barriers to timely diagnosis, access to specialist and tertiary cancer care, treatment adherence, supportive care, and the indirect costs associated with treatment, such as transportation and time away from work. Consequently, advances in NHL treatment and care may have disproportionately benefited more advantaged groups, contributing to the widening socioeconomic inequalities observed over time.

The concentration of lymphoma mortality among lower SES populations observed in this study is consistent with broader patterns of SES inequalities in cancer mortality reported in the literature. A large census-linked population-based study in Europe found that individuals with lower education had a higher cancer specific mortality rate for most cancer types, with cancer mortality in Europe mostly driven by trends in lower-education groups [16]. In terms of mortality specifically related to lymphoma, a California-based study of over 33,000 DLBCL patients found that those in lower-SES neighborhoods had approximately 24% higher lymphoma-specific mortality rates than those in higher-SES neighborhoods, with inequalities persisting despite improvements in treatment [44]. The consistency of income- and education-related gradients in lymphoma mortality across different countries and healthcare systems underscore that socioeconomic disadvantages shape lymphoma outcomes through pathways that extend beyond individual clinical factors, including barriers to timely diagnosis, equitable access to treatment, and systemic inequities in the distribution of cancer risk. Reducing these inequalities will require targeted policy interventions that address the upstream social determinants of lymphoma mortality.

### 4.3. Limitation

Several limitations should be acknowledged. First, reliance on area-level measures of income and education may not accurately capture individual socioeconomic circumstances, raising the possibility of ecological bias [45]. Second, mortality was analyzed by broad lymphoma categories (HL and NHL) without stratification by histological subtype, disease stage, or treatment modality, limiting the ability to identify socioeconomic gradients within clinically distinct subgroups. This is important given that lymphoma comprises heterogeneous malignancies with considerable variation in prognosis, treatment response, and survival across subtypes. As a result, observed socioeconomic gradients in mortality may reflect differences not only in disease occurrence, but also in survival, access to treatment, early diagnosis, and advances in therapies such as rituximab for certain lymphoma subtypes. Third, because CCP/NHS data are collected every five years, socioeconomic and demographic characteristics at the CD level were linked to the closest available CVSD year, which may introduce some temporal mismatch. Fourth, mortality trends presented in Table 1 and Table 2 were based on crude mortality rates rather than age-standardized rates. Although crude rates reflect the overall population burden of mortality, they may also be influenced by changes in the population age structure over time. Consequently, these trends should be interpreted as approximations of underlying mortality patterns rather than estimates that are entirely independent of changes in the population’s age composition. Therefore, the observed temporal trends should be interpreted with caution, as they may not solely reflect changes in age-specific mortality. Finally, the cross-sectional nature of the study precludes causal inference, allowing only the identification of associations rather than cause-and-effect relationships between SES and lymphoma mortality.

## 5. Conclusions

Over the two-decade study period, persistent and widening socioeconomic gradients were observed in HL and NHL mortality, respectively, with mortality disproportionately concentrated in lower income and educational attainment population. These findings highlight that, even within a universal healthcare system, socioeconomic disadvantage continues to shape lymphoma outcomes, likely through disparities in underlying risk factors, barriers to timely diagnosis, continuity of treatment, and access to supportive care. Addressing these inequities will require policies and interventions aimed at reducing non-medical barriers to care, including barriers to timely diagnosis, treatment continuity, and supportive services. In addition, future research should integrate individual-level socioeconomic data with detailed clinical information to better inform targeted, equity-oriented cancer control strategies.

## Figures and Tables

**Table 1:** Crude Hodgkin lymphoma mortality for both sexes per 100,000 population by Canadian region from 2000 to 2019.

Year	British Columbia	Prairies	Ontario	Eastern	Canada ^†^
2000	0.39	0.30	0.44	0.53	0.44
2001	0.26	0.50	0.44	0.48	0.44
2002	0.26	0.40	0.44	0.27	0.36
2003	0.13	0.20	0.53	0.32	0.36
2004	0.37	0.37	0.38	0.46	0.40
2005	0.37	0.28	0.42	0.62	0.45
2006	0.49	0.37	0.38	0.41	0.40
2007	0.25	0.37	0.50	0.46	0.43
2008	0.25	0.28	0.29	0.41	0.32
2009	0.24	0.26	0.43	0.45	0.38
2010	0.36	0.35	0.39	0.55	0.43
2011	0.23	0.35	0.43	0.40	0.38
2012	0.23	0.17	0.47	0.50	0.40
2013	0.35	0.26	0.39	0.30	0.34
2014	0.44	0.24	0.38	0.44	0.35
2015	0.33	0.32	0.42	0.24	0.37
2016	0.22	0.16	0.30	0.34	0.28
2017	0.33	0.24	0.30	0.44	0.34
2018	0.22	0.24	0.27	0.49	0.32
2019	0.33	0.24	0.38	0.44	0.36
Mean	0.30	0.30	0.40	0.43	0.38
Trend Coefficients	−0.0003	**−0.008**	**−0.007**	−0.002	**−0.005**
*p*-values	0.927	**0.008**	**0.009**	0.523	**0.003**

Notes: Bold font indicates statistical significance at a *p*-value < 0.05; The Prairies consist of the provinces of Alberta, Manitoba, and Saskatchewan; Eastern Canada consists of the provinces of Quebec, New Brunswick, Nova Scotia, Prince Edward Island, and Newfoundland and Labrador. ^†^ Excluding territories (Yukon, Northwest Territories, Nunavut) due to data limitations.

**Table 2:** Crude non-Hodgkin lymphoma mortality per 100,000 population by Canadian region from 2000 to 2019.

Year	British Columbia	Prairies	Ontario	Eastern	Canada ^†^
**Males**					
2000	9.72	8.83	9.13	9.25	9.20
2001	9.98	8.63	9.68	9.80	9.57
2002	9.98	8.63	9.04	9.30	9.18
2003	9.45	8.83	9.50	9.74	9.45
2004	9.76	8.09	9.44	8.65	9.00
2005	10.26	8.28	8.85	8.65	8.87
2006	10.01	8.66	8.00	8.75	8.61
2007	8.76	8.47	9.61	9.39	9.23
2008	8.76	7.71	9.19	9.91	9.10
2009	9.88	6.78	8.82	9.44	8.78
2010	8.92	6.78	8.09	9.54	8.41
2011	8.94	7.13	8.74	9.84	8.81
2012	8.70	7.48	8.90	9.84	8.91
2013	9.41	7.65	9.22	9.44	9.03
2014	8.49	6.52	9.12	10.38	8.93
2015	10.28	7.63	10.04	9.49	9.46
2016	10.50	6.83	8.34	10.97	9.13
2017	10.73	8.26	9.97	10.58	9.93
2018	9.61	6.83	9.81	10.08	9.31
2019	10.50	7.31	9.97	10.08	9.58
Mean	9.63	7.77	9.17	9.66	9.13
Trend Coefficients	0.140	**−0.095**	0.0218	**0.066**	0.012
*p*-values	0.615	**0.000**	0.362	**0.002**	0.419
**Females**					
2000	7.63	7.15	7.47	8.77	7.85
2001	6.62	7.15	7.90	7.93	7.62
2002	6.87	8.14	7.38	8.29	7.73
2003	7.89	6.75	8.25	7.56	7.73
2004	7.23	7.44	7.56	8.79	7.88
2005	5.78	6.89	7.15	7.57	7.06
2006	7.47	7.07	6.67	7.78	7.19
2007	6.98	6.89	7.80	8.38	7.72
2008	6.26	6.70	7.32	7.88	7.25
2009	7.23	6.08	6.95	7.56	7.03
2010	6.99	5.56	7.11	6.97	6.78
2011	6.82	5.04	6.72	7.66	6.73
2012	6.82	6.78	7.11	7.86	7.24
2013	6.59	6.26	7.11	8.54	7.33
2014	5.60	5.25	7.09	7.70	6.74
2015	6.24	5.57	7.46	7.70	7.03
2016	6.03	6.05	6.87	7.98	6.94
2017	7.75	5.41	7.46	7.60	7.17
2018	6.03	6.68	7.24	8.37	7.31
2019	6.46	6.05	6.57	8.08	6.91
Mean	6.77	6.45	7.26	7.95	7.26
Trend Coefficients	−0.046	**−0.095**	**−0.037**	−0.015	**−0.043**
*p*-values	0.067	**0.001**	**0.021**	0.416	**0.001**
**Both Sexes**					
2000	8.66	7.99	8.28	9.00	8.51
2001	8.27	7.89	8.77	8.84	8.58
2002	8.40	8.39	8.20	8.79	8.44
2003	8.66	7.79	8.86	8.63	8.58
2004	8.47	7.77	8.48	8.72	8.43
2005	7.98	7.58	7.98	8.10	7.95
2006	8.71	7.86	7.32	8.26	7.88
2007	7.85	7.67	8.69	8.87	8.46
2008	7.49	7.20	8.23	8.87	8.16
2009	8.53	6.43	7.86	8.48	7.89
2010	7.94	6.17	7.59	8.23	7.58
2011	7.86	6.09	7.71	8.73	7.76
2012	7.75	7.13	7.98	8.83	8.06
2013	7.98	6.96	8.14	8.98	8.17
2014	7.02	5.88	8.08	9.02	7.82
2015	8.22	6.60	8.72	8.58	8.22
2016	8.22	6.44	7.59	9.46	8.02
2017	9.21	6.84	8.68	9.07	8.53
2018	7.78	6.76	8.50	9.21	8.30
2019	8.44	6.68	8.23	9.07	8.22
Mean	8.17	7.11	8.19	8.79	8.18
Trend Coefficients	−0.017	**−0.095**	−0.008	0.025	0.016
*p*-values	0.391	**0.000**	0.642	0.052	0.178

Notes: Bold font indicates statistical significance at a *p*-value < 0.05; The Prairies consist of the provinces of Alberta, Manitoba, and Saskatchewan; Eastern Canada consists of the provinces of Quebec, New Brunswick, Nova Scotia, Prince Edward Island, and Newfoundland and Labrador. ^†^ Excluding territories (Yukon, Northwest Territories, Nunavut) due to data limitation.

**Table 3:** Age-standardized concentration index (C) demonstrating the degree of inequality in Hodgkin lymphoma mortality according to mean/median household income (equivalized) and education level (proportional attainment of a bachelor’s degree or higher) in Canada ^†^ (both sexes), from 2000 to 2019.

Year	Mean Household Equivalized Income	Median Household Equivalized Income	Education (Bachelor’s Degree or Higher)
2000	−0.0231 (−0.1248 to 0.0786)	−0.0039 (−0.1097 to 0.102)	−0.0492 (−0.1364 to 0.0380)
2001	**−0.1120 (−0.2235 to −0.0005)**	−0.0792 (−0.1984 to 0.04)	**−0.1280 (−0.2221 to −0.0339)**
2002	−0.0222 (−0.1616 to 0.1172)	0.0259 (−0.1321 to 0.1839)	−0.1010 (−0.2198 to 0.0178)
2003	0.0795 (−0.0614 to 0.2204)	0.0316 (−0.094 to 0.1572)	0.0474 (−0.0978 to 0.1926)
2004	0.0097 (−0.1269 to 0.1463)	0.0234 (−0.1326 to 0.1794)	0.0822 (−0.0405 to 0.2049)
2005	−0.0966 (−0.214 to 0.0208)	−0.0963 (−0.2188 to 0.0262)	−0.0727 (−0.1623 to 0.0169)
2006	0.0022 (−0.1087 to 0.1132)	0.0062 (−0.1239 to 0.1364)	−0.0285 (−0.1136 to 0.0566)
2007	−0.0773 (−0.1804 to 0.0258)	−0.0798 (−0.1815 to 0.0219)	−0.0837 (−0.1931 to 0.0257)
2008	**−0.1120 (−0.2233 to −0.0007)**	−0.0634 (−0.1824 to 0.0556)	**−0.1220 (−0.221 to −0.0230)**
2009	−0.1190 (−0.2409 to 0.0029)	−0.107 (−0.2254 to 0.0114)	−0.1230 (−0.2598 to 0.0138)
2010	**−0.1580 (−0.2732 to −0.0428)**	−0.0628 (−0.1849 to 0.0593)	−0.1140 (−0.2363 to 0.0083)
2011	**−0.1360 (−0.2338 to −0.0382)**	**−0.103 (−0.203 to −0.003)**	**−0.0907 (−0.1783 to −0.0031)**
2012	−0.0671 (−0.1894 to 0.0552)	−0.0249 (−0.1509 to 0.1011)	−0.0947 (−0.1919 to 0.0025)
2013	−0.0582 (−0.1919 to 0.0755)	−0.038 (−0.1630 to 0.0870)	−0.0099 (−0.1135 to 0.0938)
2014	**−0.1600 (−0.2639 to −0.0561)**	**−0.135 (−0.2242 to −0.0458)**	0.0076 (−0.0826 to 0.0977)
2015	−0.0457 (−0.1853 to 0.0939)	0.0263 (−0.1052 to 0.1578)	**−0.1390 (−0.2288 to −0.0492)**
2016	−0.0151 (−0.1305 to 0.1003)	0.0491 (−0.0544 to 0.1526)	−0.0113 (−0.1068 to 0.0842)
2017	−0.0608 (−0.1888 to 0.0672)	−0.0566 (−0.1901 to 0.0769)	−0.0204 (−0.1488 to 0.1080)
2018	**−0.1760 (−0.2877 to −0.0643)**	−0.0941 (−0.2182 to 0.03)	**−0.1620 (−0.2653 to −0.0587)**
2019	−0.0695 (−0.1881 to 0.0491)	−0.0489 (−0.1608 to 0.063)	−0.0761 (−0.1745 to 0.0223)
Trend Coefficients	−0.0041	−0.0019	−0.0016
*p*-values	0.106	0.385	0.531

Notes: 95% confidence intervals are reported in parentheses; Bold font indicates statistical significance at a *p*-value < 0.05. ^†^ Excluding territories (Yukon, Northwest Territories, Nunavut) due to data limitations.

**Table 4:** Age-standardized concentration index (C) demonstrating the degree of inequality in non-Hodgkin lymphoma mortality according to mean/median household income (equivalized) in Canada ^†^, by sex, from 2000 to 2019.

Year	Mean Household Equivalized Income			Median Household Equivalized Income		
	Males	Females	Both Sexes	Males	Females	Both Sexes
2000	−0.0304 (−0.0674 to 0.0066)	−0.0247 (−0.0655 to 0.0161)	−0.0278 (−0.0592 to 0.0036)	−0.0245 (−0.0649 to 0.0159)	−0.0169 (−0.0573 to 0.0235)	−0.0205 (−0.0519 to 0.0109)
2001	−0.0060 (−0.0358 to 0.0238)	−0.0020 (−0.0333 to 0.0294)	−0.0055 (−0.0288 to 0.0178)	−0.0098 (−0.0441 to 0.0245)	−0.0036 (−0.0383 to 0.0311)	−0.0075 (−0.0344 to 0.0193)
2002	−0.0189 (−0.0522 to 0.0144)	**−0.0670 (−0.1064 to −0.0276)**	**−0.0431 (−0.0682 to −0.0180)**	−0.0105 (−0.0477 to 0.0267)	**−0.0611 (−0.1023 to −0.0199)**	**−0.0357 (−0.0639 to −0.0075)**
2003	−0.0264 (−0.0644 to 0.0116)	0.0007 (−0.0375 to 0.0389)	−0.0146 (−0.0442 to 0.0150)	−0.0158 (−0.0575 to 0.0259)	0.0195 (−0.0175 to 0.0565)	0.0000 (−0.0321 to 0.0322)
2004	−0.0060 (−0.0526 to 0.0407)	**−0.0384 (−0.0723 to −0.0045)**	−0.0220 (−0.0524 to 0.0084)	−0.0157 (−0.0580 to 0.0266)	−0.0292 (−0.0662 to 0.0078)	−0.0215 (−0.0507 to 0.0077)
2005	0.0067 (−0.0347 to 0.0480)	−0.0219 (−0.0674 to 0.0236)	−0.0081 (−0.0416 to 0.0254)	0.0235 (−0.0188 to 0.0658)	−0.0343 (−0.0794 to 0.0108)	−0.0028 (−0.0403 to 0.0346)
2006	−0.0201 (−0.0552 to 0.0150)	**−0.0544 (−0.0871 to −0.0217)**	**−0.0389 (−0.0648 to −0.0130)**	0.0008 (−0.0365 to 0.0380)	**−0.0459 (−0.0826 to −0.0092)**	−0.0230 (−0.0516 to 0.0056)
2007	−0.0273 (−0.0608 to 0.0062)	**−0.0425 (−0.0807 to −0.0043)**	**−0.0345 (−0.0639 to −0.0051)**	−0.0111 (−0.0466 to 0.0244)	−0.0177 (−0.0606 to 0.0252)	−0.0136 (−0.0454 to 0.0182)
2008	−0.0222 (−0.0579 to 0.0135)	−0.0342 (−0.0783 to 0.0099)	−0.0289 (−0.0620 to 0.0042)	0.0050 (−0.0293 to 0.0393)	0.0057 (−0.0415 to 0.0529)	0.0052 (−0.0264 to 0.0367)
2009	−0.0225 (−0.0597 to 0.0147)	**−0.0492 (−0.0855 to −0.0129)**	**−0.0342 (−0.0638 to −0.0046)**	−0.0058 (−0.0415 to 0.0298)	**−0.0387 (−0.0738 to −0.0036)**	−0.0188 (−0.0484 to 0.0108)
2010	**−0.0519 (−0.085 to −0.0188)**	−0.0165 (−0.0567 to 0.0237)	**−0.0357 (−0.0622 to −0.0092)**	−0.0334 (−0.0681 to 0.0013)	−0.0180 (−0.0611 to 0.0251)	−0.0260 (−0.0548 to 0.0028)
2011	**−0.0445 (−0.0784 to −0.0106)**	**−0.0490 (−0.0872 to −0.0108)**	**−0.0485 (−0.0781 to −0.0189)**	−0.0279 (−0.0636 to 0.0078)	−0.0263 (−0.0651 to 0.0125)	−0.0283 (−0.0597 to 0.0031)
2012	−0.0358 (−0.0723 to 0.0007)	−0.0375 (−0.0757 to 0.0007)	**−0.0381 (−0.0691 to −0.0071)**	−0.0141 (−0.0523 to 0.0241)	−0.0076 (−0.0433 to 0.0280)	−0.0118 (−0.0420 to 0.0184)
2013	−0.0164 (−0.0478 to 0.0150)	**−0.0436 (−0.0812 to −0.0060)**	−0.0290 (−0.0580 to 0.0000)	0.0019 (−0.0261 to 0.0299)	−0.0150 (−0.0528 to 0.0228)	−0.0050 (−0.0316 to 0.0217)
2014	**−0.0755 (−0.1151 to −0.0359)**	**−0.0590 (−0.0964 to −0.0216)**	**−0.0679 (−0.0983 to −0.0375)**	**−0.0645 (−0.0998 to −0.0292)**	**−0.0458 (−0.0856 to −0.0060)**	**−0.0552 (−0.0852 to −0.0252)**
2015	**−0.0444 (−0.0877 to −0.0011)**	**−0.0554 (−0.1068 to −0.0040)**	**−0.0496 (−0.0868 to −0.0124)**	−0.0197 (−0.0624 to 0.0230)	**−0.0671 (−0.1067 to −0.0275)**	**−0.0394 (−0.0745 to −0.0043)**
2016	**−0.0687 (−0.0997 to −0.0377)**	**−0.0442 (−0.0814 to −0.0070)**	**−0.0586 (−0.0835 to −0.0337)**	**−0.0469 (−0.0834 to −0.0104)**	−0.0341 (−0.0735 to 0.0053)	**−0.0405 (−0.0721 to −0.0089)**
2017	−0.0207 (−0.0560 to 0.0146)	**−0.0362 (−0.0719 to −0.0005)**	−0.0281 (−0.0563 to 0.0001)	−0.0058 (−0.0395 to 0.0279)	**−0.0343 (−0.0672 to −0.0014)**	−0.0169 (−0.0453 to 0.0115)
2018	**−0.0341 (−0.0666 to −0.0016)**	**−0.0787 (−0.1236 to −0.0338)**	**−0.0549 (−0.0835 to −0.0263)**	−0.0132 (−0.0475 to 0.0211)	**−0.0534 (−0.1004 to −0.0064)**	−0.0300 (−0.0627 to 0.0027)
2019	−0.0244 (−0.0614 to 0.0126)	**−0.0646 (−0.1011 to −0.0281)**	**−0.0429 (−0.0705 to −0.0153)**	−0.0073 (−0.0465 to 0.0319)	**−0.0464 (−0.0805 to −0.0123)**	−0.0231 (−0.0527 to 0.0065)
Trend Coefficients	**−0.0017**	**−0.0020**	**−0.0017**	−0.0007	−0.0015	−0.0010
*p*-values	**0.028**	**0.010**	**0.002**	0.301	0.075	0.091

Note: 95% confidence intervals are reported in parentheses; bold font indicates statistical significance at a *p*-value < 0.05. ^†^ Excluding territories (Yukon, Northwest Territories, Nunavut) due to data limitation.

**Table 5:** Age-standardized concentration index (C) demonstrating the degree of inequality in non-Hodgkin lymphoma mortality according to education level (proportional attainment of a bachelor’s degree or higher) in Canada ^†^, by sex, from 2000 to 2019.

Year	Males	Females	Both Sexes
2000	−0.0076 (−0.0462 to 0.0310)	−0.0233 (−0.0641 to 0.0175)	−0.0165 (−0.0516 to 0.0186)
2001	−0.0117 (−0.0448 to 0.0214)	−0.0042 (−0.0344 to 0.02600)	−0.0111 (−0.0368 to 0.0146)
2002	−0.0176 (−0.0448 to 0.0096)	**−0.0448 (−0.0783 to −0.0113)**	**−0.0314 (−0.0516 to −0.0112)**
2003	−0.0303 (−0.063 to 0.0024)	−0.0188 (−0.0539 to 0.0163)	**−0.0267 (−0.053 to −0.0004)**
2004	−0.0147 (−0.0594 to 0.0300)	−0.0215 (−0.0556 to 0.0126)	−0.0200 (−0.0478 to 0.0078)
2005	−0.0197 (−0.0567 to 0.0173)	−0.0157 (−0.0586 to 0.0272)	−0.0207 (−0.0532 to 0.0118)
2006	−0.0191 (−0.0491 to 0.0109)	**−0.0352 (−0.0636 to −0.0068)**	**−0.0289 (−0.0505 to −0.0073)**
2007	−0.0265 (−0.0557 to 0.0027)	−0.0375 (−0.0751 to 0.0001)	**−0.0327 (−0.0596 to −0.0058)**
2008	**−0.0383 (−0.0695 to −0.0071)**	−0.0399 (−0.0860 to 0.0062)	**−0.0413 (−0.0727 to −0.0099)**
2009	−0.0281 (−0.0604 to 0.0042)	−0.0345 (−0.0700 to 0.0010)	**−0.0334 (−0.061 to −0.0058)**
2010	**−0.0352 (−0.0656 to −0.0048)**	−0.0025 (−0.0411 to 0.0361)	−0.0209 (−0.0491 to 0.0073)
2011	−0.0328 (−0.0661 to 0.0005)	**−0.0362 (−0.0705 to −0.0019)**	**−0.0368 (−0.0648 to −0.0088)**
2012	**−0.0526 (−0.0804 to −0.0248)**	**−0.0400 (−0.0761 to −0.0039)**	**−0.0487 (−0.0728 to −0.0246)**
2013	−0.0120 (−0.0426 to 0.0186)	−0.0302 (−0.0700 to 0.0096)	−0.02200 (−0.0522 to 0.0082)
2014	**−0.0489 (−0.0932 to −0.0046)**	**−0.0469 (−0.0885 to −0.0053)**	**−0.0498 (−0.0861 to −0.0135)**
2015	**−0.0466 (−0.0805 to −0.0127)**	−0.0397 (−0.1009 to 0.0215)	**−0.0449 (−0.0845 to −0.0053)**
2016	**−0.0442 (−0.0761 to −0.0123)**	−0.0310 (−0.0702 to 0.0082)	**−0.0413 (−0.0678 to −0.0148)**
2017	−0.0291 (−0.0630 to 0.0048)	−0.0125 (−0.0423 to 0.0173)	−0.0248 (−0.0520 to 0.0024)
2018	−0.0230 (−0.0557 to 0.0097)	**−0.0632 (−0.104 to −0.0224)**	**−0.0442 (−0.0711 to −0.0173)**
2019	**−0.0330 (−0.0642 to −0.0018)**	**−0.0543 (−0.0947 to −0.0139)**	**−0.0455 (−0.0727 to −0.0183)**
Trend Coefficients	**−0.0013**	**−0.0013**	**−0.0013**
*p*-values	**0.007**	**0.035**	**0.001**

Note: 95% confidence intervals are reported in parentheses; Bold font indicates statistical significance at a *p*-value < 0.05. ^†^ Excluding territories (Yukon, Northwest Territories, Nunavut) due to data limitation.

## Data Availability

The datasets analyzed in this study are not publicly available. The data were accessed through the Atlantic Research Data Centre (ARDC), part of the Canadian Research Data Centre Network (CRDCN), under an approved research project. Access to these restricted data is limited to researchers with an approved project who meet the CRDCN access requirements and are authorized to access the data within a Research Data Centre.
